# Neurotrophin, p75, and Trk Signaling Module in the Developing Nervous System of the Marine Annelid* Platynereis dumerilii*

**DOI:** 10.1155/2016/2456062

**Published:** 2016-03-16

**Authors:** Antonella Lauri, Paola Bertucci, Detlev Arendt

**Affiliations:** ^1^Developmental Biology Unit, European Molecular Biology Laboratory, 69117 Heidelberg, Germany; ^2^Institute for Biological and Medical Imaging and Institute of Developmental Genetics, Helmholtz Zentrum München, Ingolstädter Landstraße 1, 85764 München, Germany

## Abstract

In vertebrates, neurotrophic signaling plays an important role in neuronal development, neural circuit formation, and neuronal plasticity, but its evolutionary origin remains obscure. We found and validated nucleotide sequences encoding putative neurotrophic ligands (neurotrophin, NT) and receptors (Trk and p75) in two annelids,* Platynereis dumerilii* (Errantia) and* Capitella teleta* (Sedentaria, for which some sequences were found recently by Wilson, 2009). Predicted protein sequences and structures of* Platynereis* neurotrophic molecules reveal a high degree of conservation with the vertebrate counterparts; some amino acids signatures present in the annelid Trk sequences are absent in the basal chordate amphioxus, reflecting secondary loss in the cephalochordate lineage. In addition, expression analysis of NT, Trk, and p75 during* Platynereis* development by whole-mount mRNA* in situ* hybridization supports a role of these molecules in nervous system and circuit development. These annelid data corroborate the hypothesis that the neurotrophic signaling and its involvement in shaping neural networks predate the protostome-deuterostome split and were present in bilaterian ancestors.

## 1. Introduction

During vertebrate development, differentiating neurons connect to themselves and to their target cells in order to generate a functional nervous system. Neurotrophic signaling ensures correct wiring, controlling cell survival and death, differentiation, neurite outgrowth, and target innervations [1–3] (as anticipated by the Nobel Prize Winners Levi-Montalcini and Cohen in 1986 [4, 5]). Neurotrophic signaling is also active in the adult nervous system, where it is involved in learning, memory, and plasticity, modulating long-term potentiation (LTP [6, 7]). Despite the importance of the neurotrophic signaling pathway for the functioning of the vertebrate nervous system, it is only very recently that its evolutionary origin and early function started to be revealed from invertebrate data [8–13]. In vertebrates, several neurotrophin ligands (NT), such as brain derived-neurotrophic factor (BDNF), nerve growth factor (NGF), NT3, and NT4/5 (and the NGF-related NT6/7 found in fish), bind to the high affinity tyrosine kinase receptors TrkA, TrkB, and TrkC, members of the RTK (receptor tyrosine kinase) superfamily, signaling through a tyrosine kinase intracellular domain (TK). They also bind to the low affinity coreceptor p75, member of the TNRF (tumor necrosis factor receptor) superfamily, signaling through an intracellular death domain (DD). Generally, upon neurotrophin binding, the Trk receptors are autophosphorylated in their TK domain and activate MAPK/ERK, AKT, and PLC*γ* signaling (promoting cell survival, cytoskeletal rearrangement, long-term potentiation, and neuronal plasticity in the growing neural circuits [3]). When the immature form of neurotrophin (proneurotrophin) binds p75 together with the Sortilin homodimer, it induces neuronal death and controls responses to neuronal injuries [14–16]. It is clear that much of the complexity of the neurotrophic signaling has evolved in the vertebrate lineage only, in the course of the two rounds of genome duplication. The jawless lampreys, for example, seem to possess only single ancestral forms of NT and two Trk receptors [17].

Neurotrophic signaling has long been considered a vertebrate novelty; yet, the cloning and characterization of neurotrophic signaling-related molecules also in invertebrates changed this view. A conserved Trk receptor was found in the cephalochordate amphioxus [10] and in other deuterostomes [18, 19]. Several neurotrophic signaling-related molecules were also found in various protostomes (such as* Lymnaea* [8, 9] and* Drosophila* [11]), suggesting an early bilaterian origin of neurotrophic signaling. This was recently confirmed via the identification of complete Trk, p75, and NT-like genes in the genome of the crustacean* Daphnia pulex* [12] and the isolation of a functionally equivalent Trk and neurotrophin molecule in mollusks [13]. Here, we investigate candidate ligands and receptors for neurotrophic signaling in* Platynereis dumerilii*, a marine annelid that belongs to the Errantia, a group of mostly freely moving annelid worms [20]. In comparison to other protostome models,* Platynereis* has undergone less evolutionary change yet is likewise amenable to molecular and genetic techniques and experimental manipulation; it is thus especially suitable for the study of ancestral molecules and cell types [21, 22]. Indeed, sequence comparison and prediction of domains and structure reveal the presence of canonical orthologs of NT, Trk, and p75 in* Platynereis*. Among known orthologs of nonchordate invertebrates,* Platynereis* neurotrophic molecules show the highest level of amino acid identity to the vertebrate counterparts found thus far. Further, whole-mount* in situ* hybridization analysis shows that these genes are expressed in the embryonic and larval central and peripheral nervous system. Furthermore, we identified a conserved NT ortholog in* Capitella teleta*, another annelid that belongs to the Sedentaria [20], an assembly of mostly sessile families. Therefore, together with recent findings in Ecdysozoa and other protostomes [12] and mollusks [13], our data support the notion that vertebrate-type NT, Trk, and p75 molecules existed in ancient annelids and bilaterians.

## 2. Materials and Methods

### 2.1. *Platynereis dumerilii* and* Capitella teleta* Culture

*Platynereis dumerilii* embryos and larvae were obtained from an established breeding culture at EMBL Heidelberg as described previously [21]. After fertilization, the embryos were raised in plastic cups, in natural seawater at 18°C, under standard light cycle conditions.* Capitella teleta* embryos and larvae were obtained as described [25].

### 2.2. Isolation of* Platynereis* and* Capitella* Neurotrophic Orthologs

*Platynereis dumerilii* (Table 1) sequence fragments were identified from available transcriptome and genome resources (Larsson et al., unpublished) upon BLAST searches with several domains of the vertebrate homologous sequences. A cDNA library was obtained from mixed larval stages between 24 hpf and 5 dpf using the GeneRacer Advanced RACE Kit (Life Technology) and the candidate sequence fragments were amplified and extended using standard polymerase chain reaction (PCR) and rapid amplification of cDNA ends (RACE).* Capitella* genes were retrieved from available resources (JGI genome portal from the Department of Energy Joint Genome Institute, University of California [26]) and compared to previously reported ones [12]. They were experimentally validated using a cDNA library of mixed stages. Genes were amplified using a high fidelity Phusion Polymerase (Thermo Fisher Scientific) (Table 1).

### 2.3. Domain Prediction and 3D* In Silico* Modeling

Protein domains were scanned and predicted using several tools from the* Expasy suite*:* Prosite* [27],* SignalP 4.0* [28], and* ProP 1.0* [29]. Protein 3D models were predicted using* CPHmodels 3.2* [30] and* M4T* [31]. The predicted structures were visualized with* Chimera* (developed by the Resource for Biocomputing, Visualization, and Informatics, funded by the National Institutes of Health, NIGMS 9P41GM103311 [32]). For TrkA-NGF in Figure 2(c), the experimentally determined complexes in TrkA-NGF were used for comparison (DOI: 10.2210/pdb1www/pdb [23]). For p75-NT3 in Figure 2(c) the experimentally determined symmetrical complexes in p75-NT3 were used for comparison (DOI: 10.2210/pdb3buk/pdb [24]).

### 2.4. Phylogenetic Analysis

Sequence data were retrieved from* Uniprot* (The Universal Protein Resource [33]) and* NCBI* databases [34] or experimentally determined. For some genes we used the sequences indicated in Wilson, 2009 [12]. Sequence alignments were generated with* Muscle* [35] and visualized with* Geneious* [36]. Maximum likelihood (ML) trees were generated with* PhyML* [37], performing 100 bootstrap replicates with the LG substitution model.

### 2.5. Whole-Mount Single ISH Hybridization and Image Processing

Whole-mount* in situ* hybridization was performed as described [38], with slight modifications for detection of weakly expressed genes.

The full-length sequences or* Pdu*Trk, p75, and NT were cloned into PCR Topo II, and antisense RNA probes were transcribed* in vitro*. For staining of the nervous system a mouse antiacetylated tubulin (Sigma), at a dilution of 1 : 250, was used as primary antibody, while DyLight 488 anti-mouse (Jackson Laboratories) was used as secondary antibody, diluted 500 times. Images were processed with Fiji [39] or Imaris (Bitplane). Brightness and contrast were adjusted equally across the whole images. Images were cropped and processed in Illustrator to assemble figures.

## 3. Results and Discussion

### 3.1. Isolation and Characterization of* Platynereis* Trk, p75, and NT

**Figure 1:**
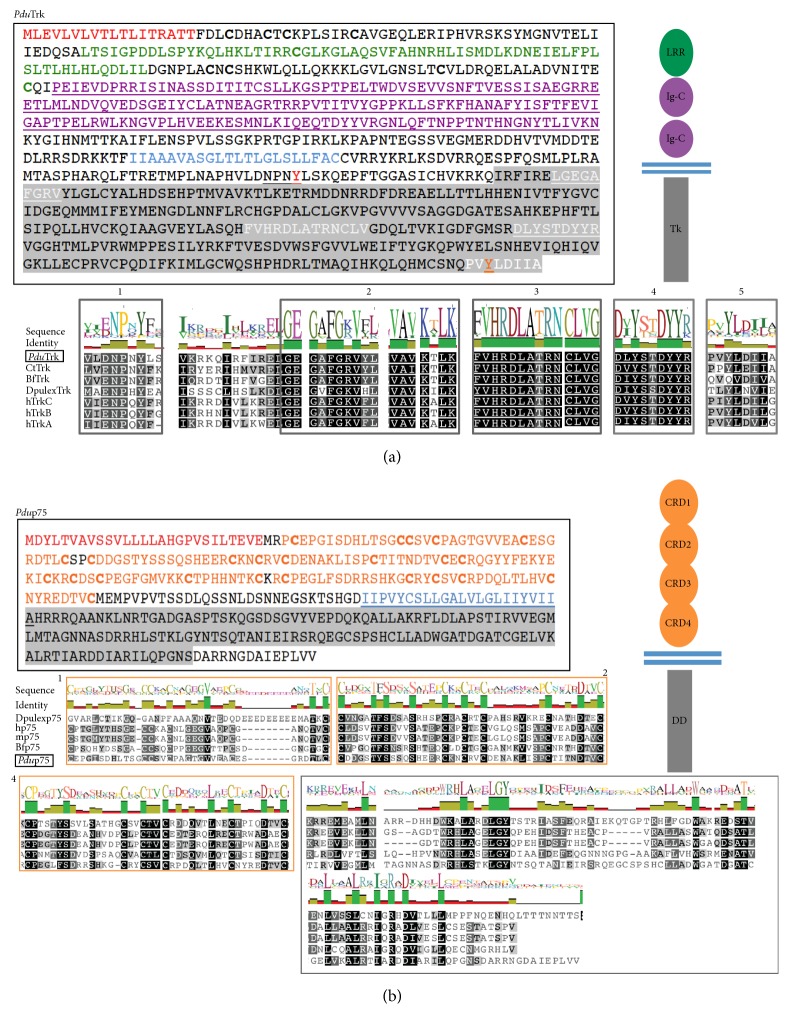
Sequence analysis of* Platynereis* Trk (*Pdu*Trk) and p75 (*Pdu*p75) shows conserved domains. (a) Predicted a.a. sequence of* Platynereis* Trk, multiple sequence alignment for* Platynereis* Trk TK intracellular domain. Predicted domains are outlined in different colors in the sequence and represented in the schematic drawing on the right. LRR, green: leucine rich repeats domains, IgG, purple: immunoglobulin domain, light blue: transmembrane domain, and TK, gray: intracellular tyrosine kinase domain. Important signatures are also highlighted in the sequence, numbered in the alignment (1–5), and described more in detail in the text. 1: juxtamembrane domain, Src binding site, 2: ATP binding site, 3: conserved aspartate (D) in the catalytic site, 4: autophosphorylation domain, and 5: binding for PLC*γ*. (b) Predicted a.a. sequence and multiple sequence alignment for* Platynereis* p75. Predicted domains: CRD1–4, orange: cysteine rich domains, light blue: transmembrane domain, and DD, gray: intracellular death domain. The alignment shows the extracellular CRD1, CRD2, and CRD4 domains and the intracellular portion, containing also the DD domain. The alignment for the less conserved CRD3 is not shown. In both red labels there is the predicted signal peptide. Pdu:* Platynereis dumerilii*, Ct:* Capitella teleta*, Bf:* Branchiostoma floridae*, Dpulex:* Daphnia pulex*, h:* Homo sapiens*, and m:* Mus musculus*.

An intracellular tyrosine kinase (TK) domain was retrieved from a* Platynereis* EST collection with several features diagnostic of Trk-like receptors (Figure 1(a), gray in the amino acids sequence, and Figure S1, in Supplementary Material available online at http://dx.doi.org/10.1155/2016/2456062): a binding domain for PTB containing proteins (1), an ATP binding site (2), a catalytic domain with the key amino acid aspartate (3), and an autophosphorylation loop (4). We found that the* Platynereis* Trk TK domain even contains a conserved docking site for the PLC*γ* [vertebrates: P(VIS)YLD(IV)L(GE),* Platynereis*: PVYLDIIA] (5 in Figure 1(a) and Figure S1(c)) that, in vertebrates, catalyses the formation of DAG (Diacylglycerol) and IP3 (Inositol Triphosphate) from PIP2 (Phosphatidylinositol 4,5-Bisphosphate) upon activation and initiates a pathway implicated in cytoskeletal rearrangement, long-term potentiation, and neuronal plasticity [40, 41]. Notably, conservation of the tyrosine for the PLC*γ* docking site is also observed in the Trk ortholog of* Daphnia pulex* (*DpulexTrk*, although the amino acids surrounding the tyrosine do not seem to be conserved in* Daphnia*, Figure 1 and Figure S1 [12]) and in* Aplysia* [13]. Conversely, this docking site is completely absent in the basal chordate amphioxus [10, 12] (Figure 1(a), Figure S1), which indicates that the amphioxus Trk ortholog has been subject to secondary loss. To determine whether* Platynereis* Trk has a chordate-like N-terminal NT-binding domain, we extended the sequence by 5′ RACE and found highly stereotypical extracellular domains (similar to* Dpulex*Trk, Figure 1(a), Figure S1). These comprised cell adhesion modules and characteristic clusters of cysteines (bold in Figure 1(a)). Beyond the signal peptide, two LRR (leucine rich repeats) domains are present (green in Figure 1(a)), followed by two predicted IgG (immunoglobulin) domains (purple in Figure 1(a)) that mediate NT binding in vertebrates [42]. The full-length version of* Platynereis* Trk shares 36,7% amino acid identity with the human TrkB, while its highly conserved TK domain shares more than 60% identity with the human TrkB (a high conservation, as compared to the Trk ortholog recently found in mollusks [13]). Thus,* Platynereis* Trk is the protostome Trk ortholog with the highest degree of amino acid identity to the vertebrate Trk counterpart. The evidences from* Daphnia*,* Aplysia*, and* Platynereis* indicate that the central core of the extracellular immunoglobulin domains was assembled before the protostome-deuterostome split, contrary to previous belief. It is plausible that* Lymnaea* lost one canonical IgG domain during evolution, due to domain reshuffling [43], but retained the ability to exert trophic functions [9].

Next, we isolated and RACE-extended the sequence of* Platynereis* p75 (Figure 1(b), Figure S2), the putative ortholog of the vertebrate low affinity coreceptor p75 that binds to the complex of Trk/NT [2, 3]. Similar to Trk,* Platynereis* p75 exhibits several conserved features (Figure 1(b) and Figure S2). The intracellular part contains a canonical death domain (DD, in the gray region highlighted in the amino acids sequence), known to activate caspases implicated in cell death and common to all the death receptors of the TNFRSF superfamily (to which p75 belongs). This domain is absent from the TNFR found in* Drosophila* (Wengen [44]). However, because the death domain is present in p75 orthologs of arthropods and other protostomes ([12] and this study), it is likely that a receptor with such domain was established already at the base of Bilateria and lost or highly modified in some lineages. Accordingly,* Drosophila* also seems to have lost a fully assembled* Trk* gene or it is not possible to recognize due to the high level of molecule divergence, similar to the presence of multiple neurotrophin molecules with a highly divergent primary sequence [11]. Four prototypical CRDs (cysteine rich domains) constitute the extracellular portion of* Platynereis* p75 (orange in Figure 1(b)), similar to p75 in vertebrates and other deuterostomes. Notably,* Daphnia* p75 harbors 3 conserved CRDs only [12].

**Figure 2:**
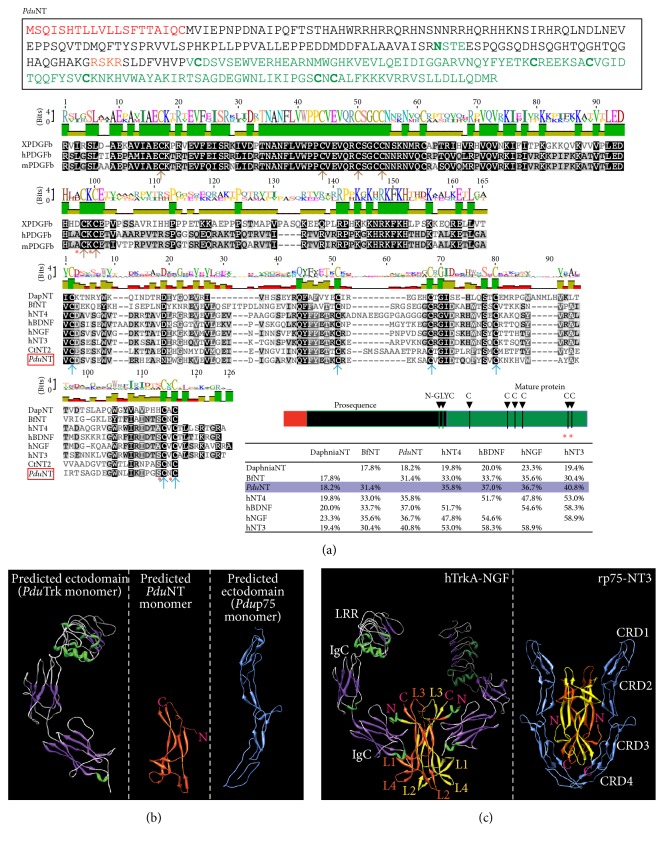
Sequence analysis of* Pdu*NT and 3D modeling of the extracellular domain of* PduT*rk, NT, and p75. (a) Predicted a.a. sequence, schematic representation, and multiple sequence alignment for* Platynereis* NT. The alignment is done for the “Cys-Knot” domain (mature protein). In comparison the Cys-Knot of PDGF-beta is also shown. In the schematics in the lower right the different domains and the cysteines core (green) are indicated. Red labels the predicted signal peptide. N-glyc: putative glycosylation site. Species are indicated as in Figure 1. In the alignment, the cysteines forming the knot are shown with arrows (brown for the PDGF subfamily, blue for the NGF subfamily). (b) Predicted 3D structure of the extracellular domain of* Pdu*Trk (left panel),* Pdu*NT (middle panel), and* Pdu*p75 (right panel). (c) As reference, a published 3D structure of the complex between the extracellular domain of TrkA-NGF (left panel, DOI: 10.2210/pdb1www/pdb [23]) and p75-NT3 (right panel, DOI: 10.2210/pdb3buk/pdb [24]) is shown. The domains are indicated (see text for details), as well as the N-terminus (N) and C-terminus (C) of the NTs (in pink), and the 4 loops formed by the NGF dimmer.

After a putative neurotrophic receptor (Trk) and coreceptor (p75) had been successfully characterized, we next set out to identify a putative candidate NT ligand. This search was more challenging as the vertebrate neurotrophins evolve relatively fast, concomitant with the extracellular domains of Trk and other RTK receptors [45]. Nevertheless, we could find one single hit for a possible NT-like molecule in the* Platynereis* transcriptome. Protein sequence analysis predicted that, similar to its vertebrate counterparts,* Platynereis* NT is composed of a signal peptide sequence comprising the first 20 amino acids and a proneurotrophin domain containing an N-glycosylation site, which is important for secretion (Figure 2(a)). Following the predicted protease cleavage site (RSKR) a core of around 120 amino acids rich in cysteines is found (Figure 2(a), green in the sequence overview). Amino acid sequence analysis and multiple sequence alignment (Figure 2(a)) show that this core of cysteines resembles the one present in the vertebrate neurotrophins (which fold into a Cys-Knot). As observed in the vertebrate neurotrophin sequences, the Cysteine “5” in the putative core of* Platynereis* NT is flanked by highly hydrophilic amino acids (serine and asparagine, red asterisks in Figure 2(a)), a key feature to predict the typical Cys-Knot foldings [46, 47]. A special motif of knotted cysteines folded by disulfide bonds is typical of growth factors and many extracellular proteins and hormones harbor such a motif (such as transforming growth factor-*β*, TGF-*β*, and platelet-derived growth factors, PDGF) [46, 48]. In these signaling molecules, such folding exposes the hydrophobic residues on the surface and helps the dimerization. Differences exist between the Cys-Knot of the different growth factor subfamilies, but all comprise 6 cysteines essential for the formation of disulfide bonds, which fold the special loops inside the *β* strands of these molecules [48], most likely an old molecular invention. The Cys-Knot of* Platynereis* NT belongs to the one shared by all neurotrophin molecules (Figure 2(a)). Further, as compared to the recently discovered* Aplysia* NT [13], we found that* Platynereis* NT shares a high amino acid identity with the vertebrate counterparts (Figure 2(a)). It shares more than 40% identity with human NT3 (in contrast to the 30% of* Aplysia* NT), 36,7% with human NGF (in contrast to the 32% of* Aplysia* NT), and 37% with BDNF (in contrast to the 28% of* Aplysia* NT). Thus,* Platynereis* neurotrophin is the most conserved protostome neurotrophin found so far.

In vertebrates, specific foldings of the Trk extracellular portion composed of IgG and LRR domains and of the p75 receptor (composed of 4 CRD) allow NT binding ([23, 24]). Thus, to further challenge sequence similarity and to assess whether* Platynereis* Trk, NT, and p75 might produce similar foldings to the vertebrate counterparts, we employed homology-based modeling algorithms and performed 3D structure prediction for* Platynereis* Trk, NT, and p75 (Figures 2(b) and 2(c)). These algorithms do not rely on a set protein or any* a priori* folding knowledge but identify and align related proteins, based on a PSI BLAST search and secondary structure prediction, upon which a 3D model is finally built. According to the homology-based 3D structure prediction, the* Platynereis* Trk extracellular domains are expected to assemble in canonical *β*-helices of the LRR and the IgG domains (Figure 2(b)), closely resembling the vertebrate situation (compare with the left panel of Figure 2(c) that shows experimentally obtained structures of human TrkA and NGF [23]).

Similarly, for* Platynereis* NT we predicted prototypical antiparallel *β*-sheets and heel-like folds that in vertebrates are determined by the Cys-Knot (Figure 2(b), middle panel). This special arrangement exposes the hydrophobic amino acids on the surface, which mediates homodimerization of neurotrophin monomers [49].

Similar to the vertebrate ortholog, our 3D modeling of the* Platynereis* p75 ectodomain predicts *β*-sheets folding into an elongated structure, with convex patches formed at the CRD2 site and between CRD3 and CRD4 (Figure 2(b), right panel); these sites have shown to be the binding patches for the neurotrophins dimmer (Figure 2(c), right panel) [24]. Our* in silico* data are only predictive and need to be validated experimentally. Biochemical analysis and crystal structures studies of the ectodomains binding to the ligands are needed to rule out whether this degree of amino acid conservation truly translates into functionally conserved domains. Nevertheless, as the models are based on primary and secondary structure alignments, these predictions are a strong indication that the extracellular domain of* Platynereis* Trk and p75 and the* Platynereis* NT possess all requisites to fold and bind to each other in a vertebrate-like fashion.

Amino acid sequence and structure prediction, as well as reciprocal BLAST, suggested that the single* Platynereis* Trk, p75, and NT proteins are orthologous to the vertebrate Trks, p75, and NTs. To confirm, we assessed the phylogenetic relationships of* Platynereis* neurotrophic signaling-related genes to their vertebrate and invertebrate counterparts.

**Figure 3:**
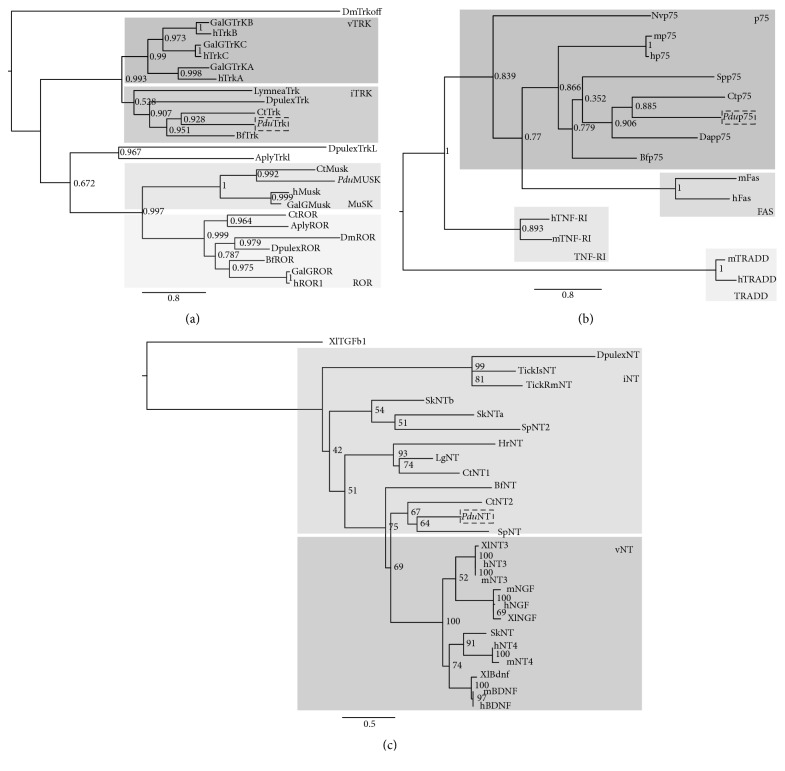
Phylogenetic analysis of annelid Trk, p75, and NT. (a) Trk; (b) p75; (c) NT. Statistical support per node is shown. Outgroups or midbranching was used to root the trees;* Platynereis* sequences are indicated with dotted black squares. ML bootstrap values are indicated for 100 replicates. For comparison, different members of the RTK (a) and TNFRSF (b) superfamily are shown (details in the text). In (a) only the tyrosine kinase domain is used for the analysis, and in (c) only the mature protein (Cys-Knot) is used, i: invertebrate, and v: vertebrates. The species used for comparison are indicated as in Figures 1 and 2. In addition, Sp:* Strongylocentrotus purpuratus*, GalG:* Gallus gallus*,* Lymnaea: Lymnaea stagnalis*, Aply:* Aplysia*, Dm:* Drosophila melanogaster*, Hr:* Helobdella robusta*, and* Nv*:* Nematostella vectensis*. TickIs:* Ixodes scapularis* tick, TickRm:* Rhipicephalus microplus* tick, and Dap or Dpulex:* Daphnia pulex*.

Maximum likelihood (ML) analysis supports orthology of* Platynereis* Trk (Figure 3(a)) that, as expected, clusters with other invertebrate Trk orthologs (“iTrk” in Figure 3(a)) and is most closely related to* Capitella* Trk. None of the invertebrate Trk sequences is more closely related to a specific vertebrate Trk type, suggesting that the latter are vertebrate-specific. Our tree likewise resolves specific clusters of Ror and Musk genes (including invertebrate members). Both Ecdysozoa (e.g.,* Daphnia*) and Lophotrochozoa (e.g.,* Platynereis* and* Capitella*) thus possess Trk, Ror, and Musk orthologs. These data support the hypothesis that the diversification of the RTK receptors into three different families (including true Trk receptors with their specific extracellular modules) predated the protostome-deuterostome split [45].

The TNFRSF superfamily contains highly divergent members, separated into different family groups [50]. Our phylogenetic analysis resolves the separation of some of the TNFRSF into separate families and supports orthology of* Platynereis* p75 with vertebrate and invertebrate p75 proteins (Figure 3(b)). From this analysis it is also apparent that the divergence of these families most likely occurred before the divergence of deuterostomes and possibly was already established in the cnidarian-bilaterian ancestor (as suggested by the presence of a* Nematostella* p75, found performing BLAST searches against the* Nematostella* database:* Nematostella vectensis* v1.0 [51]).

Higher rates of evolutionary change and shorter overall sequence lengths render the phylogenetic analysis of neurotrophins more difficult. Nevertheless, ML analysis of the predicted mature core of* Platynereis* NT confirms orthology with vertebrate neurotrophin (Figure 3(c)). We could resolve the presence of two different branches (NT3-NGF and NT4-BDNF) that likely originated at the base of the vertebrates via duplication of a single ancestral neurotrophin [52]. Among the invertebrate sequences, our analysis resolves a group of ecdysozoan NTs (including* Daphnia pulex* NT), which is distinct from that of the Lophotrochozoa and from a cluster of deuterostome NTs (the acorn worm NTs:* Sk*NTa and* Sk*NTb and* Sp*NT2) being closer to protostome rather than vertebrate homologs (confirming previous reports [12]). Further, we found and validated the presence of an additional NT in* Capitella teleta* (NT2, not reported previously). Despite the high degree of sequence divergence found in neurotrophin molecules we noted that, conversely to what has been previously reported based solely on CTN1, annelids seem to possess a more “chordate-like neurotrophin sequence” as* Platynereis* NT and the newly discovered* Capitella* NT (CTN2) fall into a group together with the chordate-like deuterostome NT of sea urchin (*Sp*NT). Thus, it is likely that a prototypic neurotrophin already existed before the protostome-deuterostome divergence. Since the genomes of* Platynereis* and* Strongylocentrotus* may still be incomplete, we cannot exclude the presence of more protostome-like (in* Platynereis*) or chordate-like (in* Strongylocentrotus*) family members. In the absence of strong support for any of the basal bilaterian nodes, the exact number of neurotrophin paralogs necessarily remains unsettled. Similar to other extracellular ligands, neurotrophins may have duplicated several times in different lineages and generated highly divergent forms (such as the Spz in arthropods [11, 12] and the apCRNF in* Aplysia* [53]), which are difficult to recognize as putative neurotrophin orthologs [45]. Furthermore, the fast evolution and divergence of neurotrophin molecules might have triggered domain reshuffling of RTK receptors [43, 45] (possibly explaining the different forms of Trk-like molecules found in invertebrates, bearing losses and gain of various domains, such as for Trkl and* Lymnaea* Trk [12]). More exhaustive ortholog searches and phylogenetic analyses in additional protostome and deuterostome genomes will be needed to clarify the early stages of neurotrophin evolution.

### 3.2. *Platynereis* NT, Trk, and p75 Are Expressed in the Developing Embryonic and Larval Nervous System

**Figure 4:**
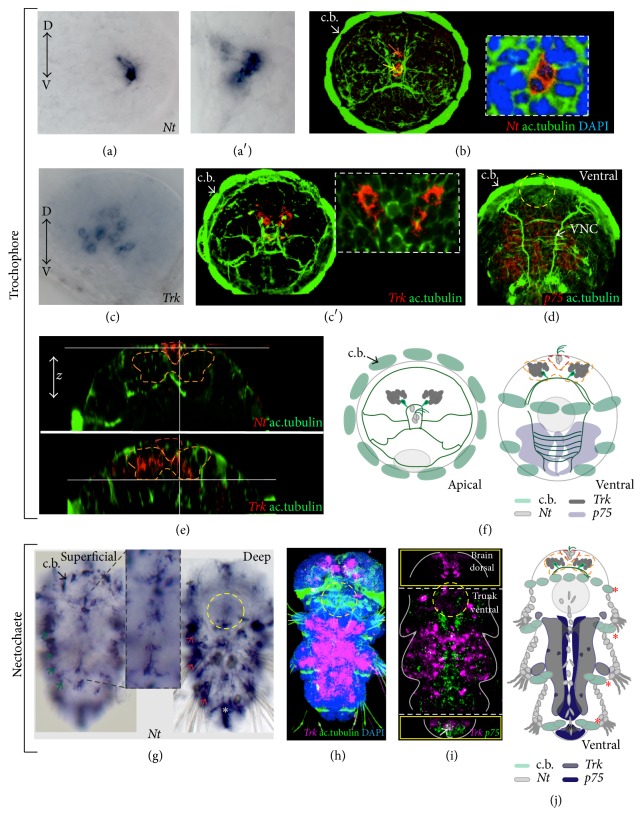
Expression of* PduNT*,* p75*, and* Trk* in the developing worm. (a–b) Apical views. Expression of* Nt* in cells of the apical organ at 48 hpf ((a′) and the inset in (b) show a close-up on the* Nt*+ cells). Bright field images (a, a′), confocal* z*-projections (b). The yellow arrow indicates the apical tuft cells; the orange arrow indicates the crescent cells. (c, c′) Apical views. Expression of* Trk* in cells in the dorsal brain (the inset in (c′) shows a close-up on the* Trk*+ cells). Bright field image (c), confocal* z*-projection (c′). (d) Ventral view. Expression of* p75* in the developing nervous system of the trunk, VNC: ventral nerve cord, confocal* z*-projection. In (a)–(d), D: dorsal, V: ventral. (e) Virtual cross sections show the position of the* Nt*+ (dashed red contour)/*Trk*+ cells (dashed orange contour) along the* z*-axis. (f) Schematic drawings of the apical view (left) and ventral view (right) showing* Nt* (light gray),* Trk* (dark gray), and* p75* (light blue) expression of the trochophore larvae. (g) Superficial and deep* Nt* expression in the juvenile larva (nectochaete, around 3 dpf). Expression in the ciliary band (c.b.) along the superficial (green arrows) and deep (red arrows) periphery and in the midline (inset in (g)) is observed. (h)* Trk* expression in the ventral trunk nervous system and in the brain in the nectochaete stage. (i) Double WMISH showing the expression of* Trk* and* p75* in the juvenile larva. The white arrow indicates colocalization at the posterior end. Different* Z*-projections from the same confocal scan are outlined by yellow squares: the upper one shows a single channel (magenta for* Trk*) within a* z*-projection of the dorsal brain (*p75* mRNA is not found here); the lower square shows the posterior growth zone. (j) Schematic drawing of the expression of* Nt*,* Trk*, and* p75* expression in the juvenile. In all the confocal images, the axonal scaffold of the nervous system is stained with acetylated tubulin (ac.tubulin, in green) and nuclei are blue (DAPI, 4′,6-diamidino-2-phenylindole). Asterisks:* Nt* expression in the pygidium (white in (g) and black in (j)); in (j)* Nt* expression in the ciliary bands (c.b., red asterisk) is indicated by a gray outline. A gray circle in (j) and a dashed yellow circle in the other panels indicate the stomodeum as reference.

To gain insight into possible sites of activity of neurotrophic signaling in* Platynereis*, we investigated* Nt*,* p75*, and* Trk* mRNA expression at several larval stages. In the early trochophore larvae,* Nt* and* Trk* mRNA were detected in the brain (Figures 4(a)–4(c′), 4(e), and 4(f)) and* p75* in the developing trunk nervous system (midline region and the left and right trunk, Figures 4(d) and 4(f)). We identified the cells producing NT (Figures 4(a)–4(b), 4(e), and 4(f)) as the apical tuft cells (yellow arrow in Figure 4(b)) and the crescent cells (orange arrow in Figure 4(b)) that are part of the apical organ. The apical organ differentiates first in the larval brain and is later complemented by developing adult brain parts [54]. Cells expressing* Trk* were found deeper in the dorsal developing adult brain (Figures 4(c), 4(c′), 4(e), and 4(f)), in cells that form part of a nonvisual, light sensitive region, which comprises vertebrate-type ciliary photoreceptors [21], produces melatonin, and harbors a circadian clock [55]. This early* Nt* and* Trk* expression suggests that the early forming apical organ emits neurotrophic signals that are received by cells of the later forming ciliary photoreceptor region and that* Platynereis* Trk might be involved in circadian clock entrainment (as reported for vertebrate TrkB [56]).

At later larval stages (referred to as nectochaete [57]), when the trunk nervous system and musculature differentiate,* Platynereis Nt* expression was still observed in the apical organ region, but additional expression sites became apparent in the trunk. Those included the ciliary bands, the ventral midline, and peripheral superficial and deep cells (green and red arrows in Figure 4(g), schematics in Figure 4(j)), likely including also nonneuronal cells (such as prospective skin cells and developing muscle cells). At the same stages,* Platynereis Trk* is expressed broadly in the developing nervous system (Figures 4(h)–4(j)). Double WMISH revealed that while* Trk* and* p75* clearly share a domain of expression at the posterior growth zone of the developing worm (white arrow in Figure 4(i)), their expression is almost mutually exclusive in the anterior nervous system. Here,* Trk* is expressed more broadly, at the left and right site of the neural tissue, while* p75* is expressed mostly in cells surrounding the midline, where* Nt* is found (Figures 4(g)–4(j)). The more restricted expression of* p75* around the site of NT secretion suggests that p75-NT might signal together independently from Trk, as reported from vertebrates. In* Platynereis* proliferating neuronal progenitors are found mostly on the surface of the ectoderm, in the medial-most domain around the wnt4+ midline (NT+), while differentiated neurons are located more laterally and basally [58]. It would be interesting to test whether NT secreted from* Platynereis* midline acts together with p75 in the nearby territory during neuronal progenitor proliferation, while* Platynereis* Trk signaling might be more predominant in differentiated neurons. For instance, in the vertebrate embryo target cells secrete neurotrophic ligands that attract the* Trk*+ axons of developing neurons, thus promoting innervation and also providing signals for neuronal survival [1].* Nt*+ cells in* Platynereis* apical organ, neuronal midline, skin, and muscles might accordingly represent also a central and peripheral target sites for the outgrowing axons of developing* Trk*+ neurons, consistent with a role of the annelid neurotrophic signaling in axon pathfinding.

### 3.3. The Evolution of the Neurotrophic Signaling in Eumetazoa

While it is clear that Trk, NT, and p75 were distinctly present and that the original tyrosine kinase had already split into different subfamilies in bilaterian ancestors (such as Trk, Ror, and Musk shown in Figure 3(a) [45]), the situation is less clear for bilaterian outgroups. According to Sossin [45], a common precursor molecule for Trk/Musk/Ror possessing a Frizzle/Kringle extracellular domain (similar to the ones present in Ror and Musk and in sponge tyrosine kinase receptors) and a conserved intracellular tyrosine kinase was likely present at the base of Eumetazoa. The split of the three families occurred only at the base of the Bilateria. In line with this, a Trk receptor and neurotrophin ligands appear to be absent from* Nematostella* ([59] and Antonella Lauri, unpublished), which seems to have only a p75 molecule (Figure 3(b)). Thus, a* bona fide* Trk and NT with their full complement of extracellular domains appear to have been evolved in the bilaterian stem line only (in contrast to p75, Figure 5).

## 4. Conclusions

In conclusion, our data on the highly conserved annelid neurotrophic molecules corroborate recent hypotheses that vertebrate-like representatives of neurotrophic signaling molecules existed at the base of Bilateria, predating the protostome-deuterostome split. The identification of a p75 ortholog in* Nematostella* furthermore indicates that this receptor was already present in the cnidarian-bilaterian ancestor. Urbilaterian neurotrophic signaling thus included a Trk receptor with a typical extracellular domain, as found in some extant, slow-evolving invertebrates (such as* Platynereis*) and in vertebrates, a p75 coreceptor, and one or more NT ligands, possibly performing trophic functions in the nervous system.

## Supplementary Material

The Supplementary material provides detailed information on the full-length sequence of *Platynereis *Trk and p75, and on the amino acid identity as compared to other species.

## Figures and Tables

**Figure 5:**
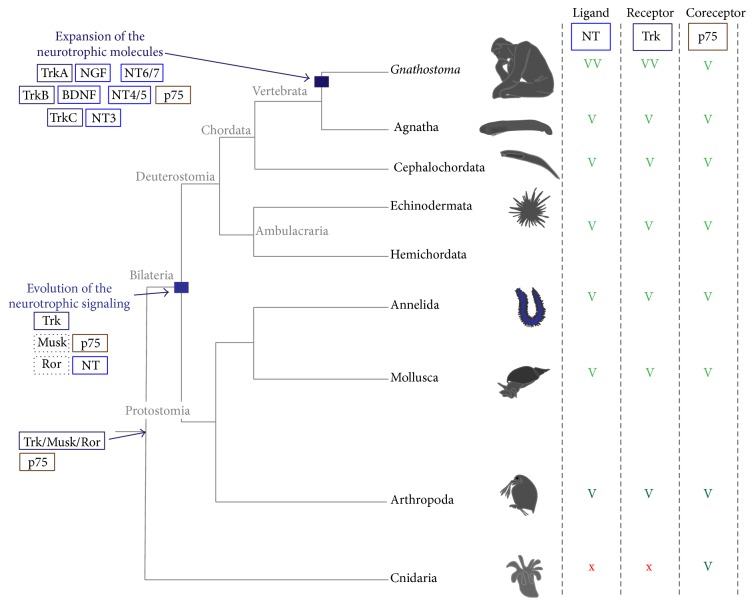
Evolution of the neurotrophic signaling predated the emergence of chordates. Eumetazoa tree showing the occurrence of NT, Trk, and p75 and the possible split of Trk from an ancestral Trk/Musk/Ror ancestor gene (after Sossin, 2006). A light green “V” is given when a conserved version of the protein is found; a dark green “V” indicates the presence of less conserved molecules (see text for details). Double light green “VV” indicates expansion of the molecules due to genome duplication. A red “x” is given when a putative ortholog is not found. Evolution of the neurotrophic signaling is hypothesized to have occurred at the base of Bilateria, while expansion of the single molecules likely occurred only in the vertebrate lineage.

**Table 1:** 

Gene	Forward primer	Reverse primer
**Pdu Trk** **(**GenBank: KU206573)	**5**′**R_A**: GGCACCTTTCCCAGACACAGGGCATCAGGGCC, **5**′**R_B**: GCAGACTCCATAAAATGTCACAATATTCTCGTG, **5**′**R_C**: CAAGGTACACTCTACCAAAAGCCCCTTCTCCTAATTC, **5**′**N_C**: TCCTAATTCTCGAATAAATCTGATCTGCTT, **5**′**R_D**: CAGGTAATTCGGATTATCCAACACATGAGG, **5**′**N_D**: ACATGAGGTGCATTTAAGGGCATGGTC, **5**′**R_E**: TCTTGGATCAACCTCAATTTCAGGGATTTGACA	ATAAGAGACAGTAATCCGTAATTAAGCAATGA

**Pdu Nt** **(**GenBank: KU206574)	GACGGAGGCTGGTCGCAAAAAACATGTCAC	GGGGGTATCACCGCATATCTTGCAGCAA

**Pdu p75** **(**GenBank: KU206572)	**5**′**R**: AGTAATACCCCTGCCGACATTCGCAGACT, **5**′**N**: GCAGACTGTGTCGTTAGTTATCGTACAGGG	**3**′**R**: GCTTCATGTGTGCAACTACAGAGAGGATAC, **3**′**N**: GATACAGTGTGTATGGAAATGCCCGTCCCAG

**C.t. Trk**	ATGTTTTTGAGTGACGTTGCGTGCT	ATCGGCGATGATTTCCAAATATGGTGG

**C.t. Nt1**	ATGCAGCTTGATTGCTGGC (scaffold 4)	AGTCAGAGTTGCGGTACAGCA

**C.t. Nt2** **(**GenBank: KU206575)	ATCGACATGCAGTGGAATCAAAGAAAATCC (scaffold669)	ATTTTCCATCAACTGAAATCGATCAGAC
