# Supplementary material for: Neurotrophin, p75, and Trk Signaling Module in the Developing Nervous System of the Marine Annelid Platynereis dumerilii
Source: Biomed Res Int. 2016 Mar 16;2016:2456062. doi: 10.1155/2016/2456062 (PMC4812194; doi:10.1155/2016/2456062)
Supplement: Supplementary file 1 — The Supplementary material provides detailed information on the full-length sequence of Platynereis Trk and p75, and on the amino acid identity as compared to other species. [file 2456062.f1.pdf]

Supplementary Material

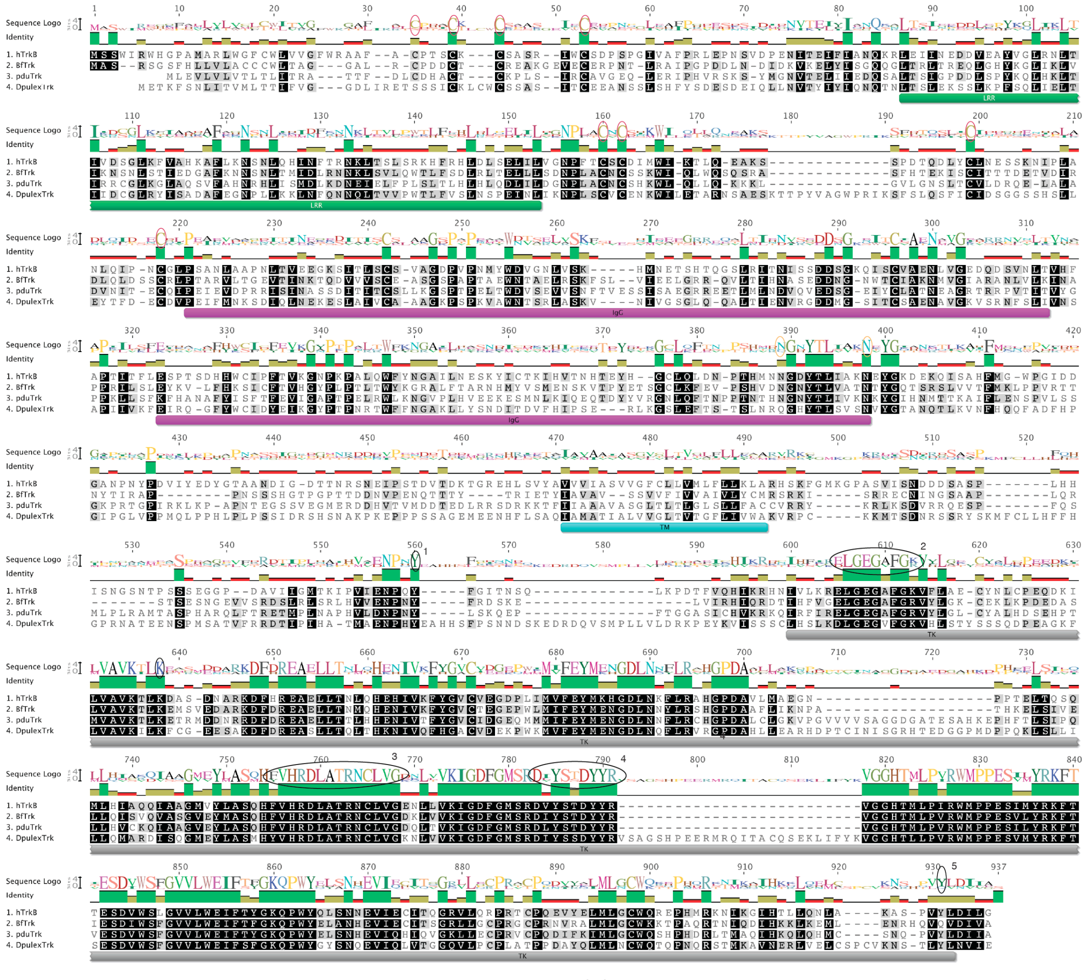

(a)

Full length

	DpulexTrk	pduTrk	BfTrk	hTrkA	hTrkB	hTrkC
DpulexTrk		32.2%	32.0%	30.4%	31.7%	31.8%
pduTrk	32.2%		38.9%	35.3%	36.7%	35.6%
BfTrk	32.0%	38.9%		38.8%	39.5%	39.3%
hTrkA	30.4%	35.3%	38.8%		47.4%	48.0%
hTrkB	31.7%	36.7%	39.5%	47.4%		52.7%
hTrkC	31.8%	35.6%	39.3%	48.0%	52.7%	

TK domain

	DpulexTrk...	hTrkA TK	hTrkB TK	hTrkC TK	BfTrk TK	pduTrk Tk
DpulexTrk TK		50.1%	49.9%	49.9%	48.1%	48.8%
hTrkA TK	50.1%		76.3%	72.5%	62.8%	57.7%
hTrkB TK	49.9%	76.3%		78.5%	64.2%	61.0%
hTrkC TK	49.9%	72.5%	78.5%		59.7%	57.1%
BfTrk TK	48.1%	62.8%	64.2%	59.7%		64.7%
pduTrk Tk	48.8%	57.7%	61.0%	57.1%	64.7%	

(b)

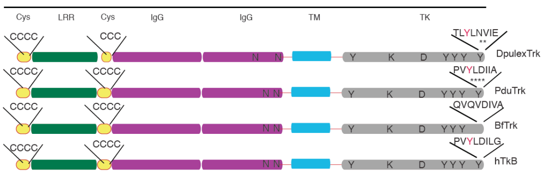

(c)

Figure S1. Analysis of the full-length amino acid sequence of *Platynereis* Trk (*Pdu*Trk) and conserved domains. (a) Multiple amino acids sequence alignment for the full length *Platynereis* Trk. Predicted domains are indicated below the sequences. LRR, green: Leucine reach repeats domains, IgG, purple: immunoglobulin domain, TM, light blue: transmembrane domain, TK, gray: intracellular tyrosine kinase domain. Conserved amino acids and signatures are highlighted. Different conserved signatures of the Tk domain are numbered in the alignment, as in fig.1a. **(b**) **I**dentity matrix showing the amino acids identity (%) for the full length (upper panel) and for the intracellular (lower panel) amino acid sequence of *Platynereis* Trk relative to the ortholog receptors of the species used in the alignment**:** Pdu (*Platynereis dumerilii*), Bf (*Brachiostoma floridae*), Dpulex (*Daphnia pulex*), h (*Homo sapiens*). **(c**) Schematics of the domains found in *Platynereis*, in comparison with *Daphnia* Trk, human

TrkB, amphioxus Trk. Conversely to amphioxus, *Platynereis* PLCγ docking site (PVYLDIIA) is highly conserved. The important tyrosine of this signature is highlighted in red. Asterisks indicate the amino acid conservation around the Y.

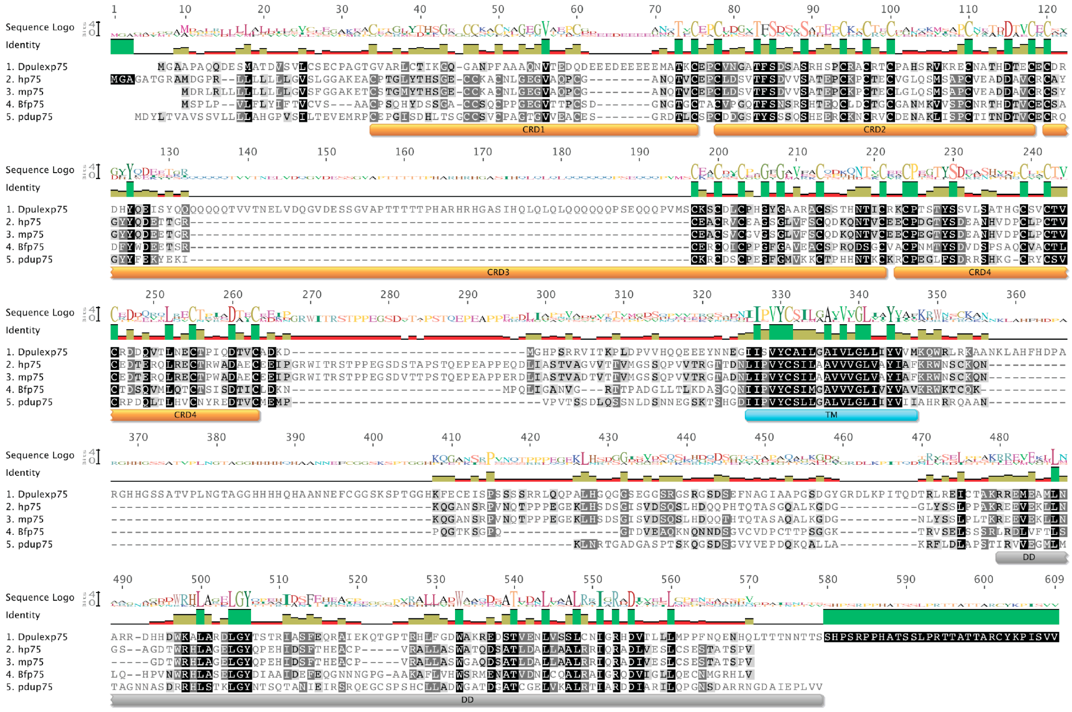

(a)

	Dpulexp75	hp75	mp75	Bfp75	pdup75
Dpulexp75		17.6%	17.5%	21.4%	20.1%
hp75	17.6%		92.1%	31.2%	23.9%
mp75	17.5%	92.1%		31.3%	25.4%
Bfp75	21.4%	31.2%	31.3%		29.3%
pdup75	20.1%	23.9%	25.4%	29.3%	

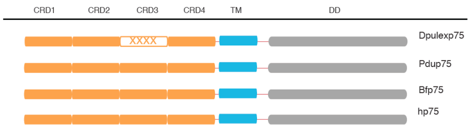

(b) (c)

Figure S2. Analysis of the full-length amino acid sequence of *Platynereis* p75 (*Pdu*p75) and conserved domains (a) Multiple amino acids sequence alignment for the full length of *Platynereis* p75. Predicted domains: CRD1-4 (cystein rich domain), orange. TM, light blue: transmembrane domain; the intracellular domain (grey) bearing the dead domain (DD). **(b)** Identity matrix showing the amimo acids identity (%) for the full length amino acid sequence of *Platynereis* p75 relative to the ortholog receptors of the species used in the alignment**:** Pdu (*Platynereis dumerilii*), Bf (*Brachiostoma floridae*), Dpulex (*Daphnia pulex*), h (*Homo sapiens*), m (*Mus musculus*).(**c**)Schematics of the domains found in *Platynereis* as compared to *Daphnia*, human Trk. Note that a 3rd CRD is absent in *Daphnia* sequence.
